# Exome sequencing reveals a high genetic heterogeneity on familial Hirschsprung disease

**DOI:** 10.1038/srep16473

**Published:** 2015-11-12

**Authors:** Berta Luzón-Toro, Hongsheng Gui, Macarena Ruiz-Ferrer, Clara Sze-Man Tang, Raquel M. Fernández, Pak-Chung Sham, Ana Torroglosa, Paul Kwong-Hang Tam, Laura Espino-Paisán, Stacey S. Cherny, Marta Bleda, María del Valle Enguix-Riego, Joaquín Dopazo, Guillermo Antiñolo, María-Mercé García-Barceló, Salud Borrego

**Affiliations:** 1 Department of Genetics, Reproduction and Fetal Medicine, Institute of Biomedicine of Seville (IBIS), University Hospital Virgen del Rocío/CSIC/University of Seville, Seville, Spain; 2 Centre for Biomedical Network Research on Rare Diseases (CIBERER), Spain; 3 Centre for Genomic Sciences, Li Ka Shing Faculty of Medicine, The University of Hong Kong, Hong Kong, China; 4 Department of Psychiatry, Li Ka Shing Faculty of Medicine, The University of Hong Kong, Hong Kong, China; 5 Department of Surgery, Li Ka Shing Faculty of Medicine, The University of Hong Kong, Hong Kong, China; 6 State Key Laboratory of Brain and Cognitive Sciences, Li Ka Shing Faculty of Medicine, The University of Hong Kong, Hong Kong, China; 7 Centre for Reproduction, Development, and Growth, Li Ka Shing Faculty of Medicine, The University of Hong Kong, Hong Kong, China; 8 Computational Genomics Department, Centro de Investigación Príncipe Felipe (CIPF), Valencia, Spain; 9 Functional Genomics Node, (INB) at CIPF, Valencia, Spain

## Abstract

Hirschsprung disease (HSCR; OMIM 142623) is a developmental disorder characterized by aganglionosis along variable lengths of the distal gastrointestinal tract, which results in intestinal obstruction. Interactions among known HSCR genes and/or unknown disease susceptibility loci lead to variable severity of phenotype. Neither linkage nor genome-wide association studies have efficiently contributed to completely dissect the genetic pathways underlying this complex genetic disorder. We have performed whole exome sequencing of 16 HSCR patients from 8 unrelated families with SOLID platform. Variants shared by affected relatives were validated by Sanger sequencing. We searched for genes recurrently mutated across families. Only variations in the *FAT3* gene were significantly enriched in five families. Within-family analysis identified compound heterozygotes for *AHNAK* and several genes (N = 23) with heterozygous variants that co-segregated with the phenotype. Network and pathway analyses facilitated the discovery of polygenic inheritance involving *FAT3*, HSCR known genes and their gene partners. Altogether, our approach has facilitated the detection of more than one damaging variant in biologically plausible genes that could jointly contribute to the phenotype. Our data may contribute to the understanding of the complex interactions that occur during enteric nervous system development and the etiopathology of familial HSCR.

Hirschsprung disease (HSCR, OMIM 142623) is a developmental disorder occurring in approximately 1 of 5,000 live births^1^. HSCR mostly presents sporadically, with only 5–20% of cases being familial, and manifests with low, sex dependent penetrance and variable expression of phenotype. It is characterized by the absence of ganglion cells along variable lengths of the distal gastrointestinal tract, which results in tonic contraction of the aganglionic colon segment and functional intestinal obstruction. Such aganglionosis is associated with a delay in the entry of neural crest-derived cells into the foregut, as well as a delayed progression of enteric neural crest cells along the gut^2^,^3^.

The genetic aetiology of the disease is likely to be heterogeneous, and involving common and rare variants acting alone or in combination^4^. For only a small fraction of HSCR patients, the phenotype is caused by unique damaging mutations in coding sequences of genes encoding protein components of signalling pathways involved in the development of the enteric nervous system (ENS), with the *RET* proto-oncogene being the most important. *RET* coding rare mutations account for up to 50% of the familial cases and 10–20% of the sporadic cases. About 5% of the cases are due to mutations in genes other than *RET*, namely *GDNF*, *NRTN*, *PSPN*, *EDNRB*, *EDN3*, *ECE1*, *NTF3*, *NTRK3*, *SOX10, PHOX2B*, *L1CAM*, *ZFHX1B, KIAA1279*, *TCF4*, *PROK1*, *PROKR1*, *PROKR2*, *GFRA1*, *NRG1*, *NRG3*, *Class 3 SEMAPHORINs* (*3A*, *3C* and *3D*) and *DNMT3B*, which are known to play an important role in the development of the ENS^2^,^5^,^6^,^7^. Furthermore, a specific *RET* haplotype was identified clearly associated with the sporadic forms of HSCR, characterised by the presence of a common *RET* variant (rs2435357; 10:g.43086608T > C) located in a gut-specific *RET* enhancer element in intron 1, which it has been demonstrated to disrupt binding of *SOX10*^8^,^9^,^10^,^11^,^12^. In addition, the interaction between *RET* rs2435357 and *NRG1* rs7835688 (8:g.32531041C > G) variants was detected in Chinese HSCR patients^13^. Recently, a study has established those *RET* variants, rs2435357 and rs2506030 (10:g.42952399A > G), and *SEMA3A* variant, rs11766001 (7:g.84515886A > C), as common susceptibility alleles in HSCR subjects^14^.

The complexity of the disorder is also evident in those families where the segregation of the HSCR phenotype does not follow any recognizable pattern of inheritance, shows intrafamilial variability or affected individuals do not carry mutations in any of the known HSCR genes. Such patterns might be explained by the joint effect of more than one variant either in coding or non-coding sequences^15^. The emergence of next-generation sequencing (NGS) could help us to decode the complex genetic background of HSCR. However, to unmask the underlying variants is indeed a challenging effort where making sense of NGS data requires cutting edge bioinformatic approaches. One possible solution is the combination of whole exome sequencing (WES) together with interaction network and pathway analyses, which allow prioritizing candidate genes with functional relationship^16^ and constitute an integrated and comprehensive approach in exploring a disease in the post-genomic era. Therefore, these analyses play an increasingly important role in the diagnosis of complex and polygenic disorders^17^.

Here we present the first exome sequencing study of familial HSCR from a Caucasian population, including 8 Spanish families with 2 HSCR patients per family. Family studies provide an opportunity to explore and interpret the as-yet-unidentified genetic variation underlying many complex genetic diseases, such as familial HSCR.

## Results

**Figure 1:**
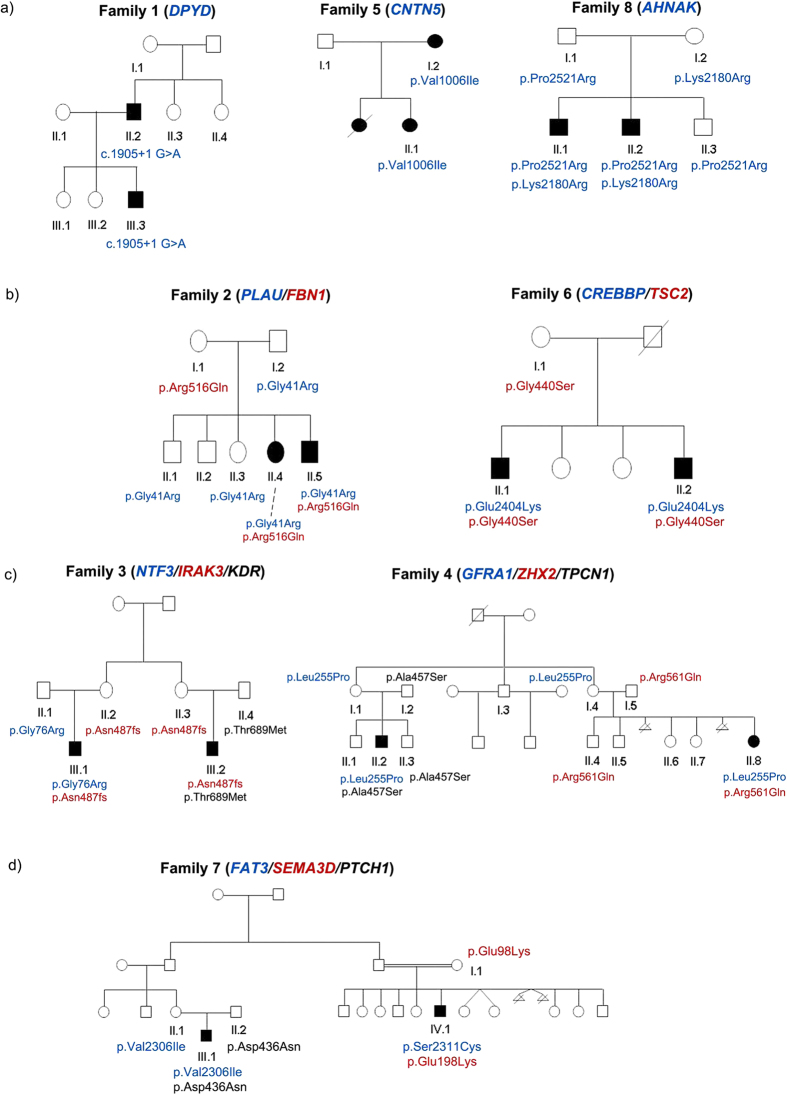
The pedigrees of the eight families affected by Hirschsprung disease included in the study. Mutational events that may explain the phenotype in each pedigree are listed. Only those individuals with pedigree identifier were sequenced. Genes and their corresponding variants are represented by different colors.

Forty-eight individuals, including 8 families with 2 patients each (Table 1 and Fig. 1), were successfully whole exome sequenced using the SOLID 5500xl platform. The average read depth achieved for target regions was 34.9X. Although it only achieved on-average 76.9% of targeted regions with read depth >10 fold, this metric increased to 85.7% when considering regions covered by 4 or more sequence reads (Supplementary S1 Table). Quality metrics for clean variants in exonic regions were within the normal range (dbSNP137 coverage >95%, Ti/TV > 3; S1 Table). Pairwise identity by descent (IBD) verified the pedigree structure (Supplementary S1 Table).

These 8 families had previously been screened for mutations in known HSCR genes by Sanger sequencing. All five rare damaging mutations identified were confirmed by WES (Table 1). After variant filtering, an average of 160 (standard deviation = 31) rare damaging variants was detected in each HSCR patient (Table 2). Variants were prioritized according if they were shared within families and/or linked to any of the 19 HSCR genes through protein-protein interaction (PPI) network or pathway analyses. For each family, the number of variants present in both affected individuals would depend on their kinship relationship (Table 2 and Fig. 1). For example, within family 7 there were only two missense single nucleotide variants (SNVs) shared because the affected family members were actually fourth-degree relatives. In contrast, affected individuals of family 8 shared 90 variants (11 as loss of function mutations, LOF) since they were siblings. A total of 246 variants potentially involved in the disease were selected for validation by Sanger sequencing. This validation verified the 89.8% of our selected rare damaging variants.

In an attempt to uncover possible causal variants we checked for cosegregation of variants with the phenotype according to the pedigree structure. Complete segregation of variants with the phenotype was only observed for families 1, 5 and 8. A few heterozygous variants fully cosegregated with the phenotype (possibly explaining the affected individuals in each family) in families 1 and 5 (Tables 2 and Supplementary S2 Table). Interestingly, two LOF mutations (in *DPYD* and *QTRTD1)* fully cosegregated with HSCR in family 1, but none LOF found for family 5. We prioritized *DPYD* in family 1 and *CNTN5* in family 5 (Fig. 1A) based on their described functional involvement in neuronal development. Affected individuals of family 8 were compound heterozygous for *AHNAK*, each different variant having been inherited from each unaffected parent (Fig. 1A). However, these genes did not carry any rare damaging variant in the remaining families.

To assess whether there was a susceptibility gene contributing to HSCR common to all these families, we performed linkage and rare variant association tests for genes that were mutated in at least two families. A total of nine genes were included (Table 3). This rare variant association test showed *FAT3* as the only significantly gene associated with the HSCR phenotype (p < 0.05 after Bonferroni correction). Subsequent comparison of *FAT3* enrichment between HSCR families and MGP controls also revealed significant difference (SKAT p-value <s3.65e-06), but not between MGP controls and 1304 public controls (VAAST p-value = 0.07).

A LOD score of 1.25 also suggested a moderate genetic linkage across families. Indeed, different *FAT3* variants were found in 5 families (Table 4). The variants were not necessarily shared by the affected members within a family. Only affected members of families 2, 3 and 6 shared the same *FAT3* variant (Genotypes of individuals with variants within *FAT3* can be found in Supplementary S2 Table).

**Figure 2:**
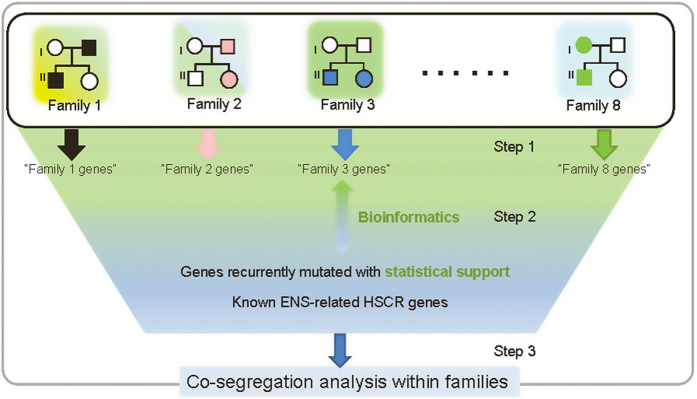
Overall analytical pipeline for gene prioritization. Pedigrees are symbolic only. Three steps were adopted to search for genes (or gene sets) that may explain HSCR families. Step 1: genes shared by two patients in the same family at the same variant site were extracted; Step2: these genes were clustered or linked to themselves, genes recurrently found with statistical support (pVAAST) and genes know to ENS development through bioinformatics analyses, within and across families; Step3: genes and gene combinations were checked for family cosegregation with the phenotype.

Data from those families carrying variants in *FAT3* (2, 3, 4, 6 and 7) were re-analyzed through PPI network and biological pathways to pinpoint additional genes that could account for the incomplete segregation of *FAT3* or other ENS genes with HSCR (Fig. 2). Variants in the two related genes, *PLAU* and *FBN1*, co-existed in patients of family 2, likewise for variants in *CREBBP* and *TSC2* in patients of family 6 (Fig. 1B). These co-existing variants had been inherited from each unaffected parent. Genes linked to the ENS were identified in family 3 (*NTF3, IRAK3* and *KDR*) and in family 4 (*GFRA1*, *ZHX2* and *TPCN1*) jointly cosegregating with the phenotype (Fig. 1C). Interestingly, although each patient of family 7 had two related genes mutated, these differed between patients (III1:*FAT3/SEMA3D*; IV1: *FAT3/PTCH1*) Moreover, the *FAT3* variants were also at different sites. Thus, a clear pattern of polygenic inheritance was observed in family 7, for which *FAT3* (two different variants)*, SEMA3D* and *PTCH1* genes seem to be necessary to explain the two affected patients (Fig. 1D). Detail of joint cosegregation pattern for each family was included in Supplementary S2 Table. No single pathway seemed to be disrupted across families.

## Discussion

As disease-associated common variants fail to provide reasonable genetic mechanism for most common disorders, rarer or structural variants or low frequency variants with intermediate effect have been proposed to explain the missing heritability^18^,^19^. Rare damaging variants usually have higher penetrance and confer larger effects on an individual’s risk of developing disease than common variants do. However, as each one of them is negatively selected and eventually disappear, such variants need to be generated (*de novo*) and accumulated to keep an overall consistent effect at population level^20^. Study designs involving >=2 cases in one or more families greatly reduce the difficulty of finding disease-causing variants among the huge amount of genomic data generated by WES analyses^21^. This strategy has been proved efficient and effective for uncovering causal genes for a few Mendelian disorders. Regarding HSCR, Yang *et al.* firstly adopted exome sequencing on a Chinese family and reported CDS mutations in *NRG3*, a gene in which copy number variations had previously been associated with the disorder^22^,^23^.

Previous studies have suggested how complex interactions among known HSCR genes or still unknown susceptibility loci lead to incomplete penetrance and variable severity of disease^24^. Although no shared *RET* rare damaging mutations were found in any of these 8 families, we cannot rule out the contribution of this gene given the demonstrated contribution of the common *RET* intron 1 risk allele (rs2435357T) to HSCR^10^. Homozygous genotype (T/T) was present in 37.5% of our patients. Indeed, linkage analyses have revealed significant allele sharing in 10q11 region yet no coding sequence *RET* mutations have been identified^24^. With this information, we speculate that the presence of the common *RET* risk allele may have increased the susceptibility to develop HSCR in some of our families^10^.

We have performed the first WES on Caucasian familial HSCR devoid of obvious mutations in the main HSCR genes. This has enabled us to systematically narrow down gene lists from all rare damaging coding sequence variants identified. We highlighted the presence of *FAT3* gene, because it was the only one with overrepresented rare damaging variants (6 variants from 5 out of 8 families). All six variants detected in *FAT3* belong to cadherin domains of the protein. Cadherins are a family of molecules that mediate Ca^2+^− dependent cell-cell adhesion and thus modulate a wide variety of processes such as cell polarisation and migration^25^. They also play a role in the interactions between neurites derived from specific subsets of neurons during development^26^,^27^. Among all the gene ontology classifications where *FAT3* is implicated, we emphasized its role in the cell morphogenesis in neuron differentiation^28^. In addition, *FAT3* gene is expressed in embryonic stem cells^29^. Mutations within this gene may lead to abnormal development of neural crest derived stem cells^30^. All these features, together with its occurrence in most of our families, suggest the implication of this gene in the aetiology of HSCR.

A large amount of literature has been published to support the interaction of pairs of genes for HSCR^6^,^13^,^16^, and this inspired us to interpret non-monogenic HSCR families by a combination of genes. We have specially focused on those genes with a role in neural development and on those that could be related with the disease through a specific process. The presence of genes previously associated to HSCR in our candidate gene list reinforces the validity of our method to detect potential genes related with the disease. The gene-pairs that came up in our analysis are known to interact because they belong to the same interacting network or pathway for enteric nervous system development. In family 7 we detected variations in *SEMA3D* and in another interesting gene, *PTCH1*, in which common variations have been already associated with HSCR in a pathway-based epitasis analysis^31^. Regarding family 4, *GFRA1* was altered together with *ZHX2* and *TPCN1*. The *ZHX2* gene, a novel regulator of neural progenitor cell maintenance, is specifically expressed in neural progenitor cells during cortical neurogenesis^32^. *In silico* analysis indicates that *TPCN1* gene is functionally related with *PTCH1*^33^. Thus, we speculate that variations in this gene could be associated with the alterations occurring during embryonic development that lead to HSCR phenotype. In family 3, we found rare damaging variants in *KDR* and *IRAK3* genes, in addition to variants in *NTF3* and *SEMA3D*. The *KDR* gene encodes the main receptor of VEGF in blood vessels, which is a growth factor for endothelial cells implicated in processes such as cellular proliferation, differentiation, survival and migration^34^. Recently, it has been associated with neuronal survival^35^ and this function could potentially link this gene with the etiopathogenesis of HSCR. The *IRAK3* gene has been associated with necrotizing enterocolitis, which has been shown to exist in specific subgroups of HSCR patients and it determines severe long-term sequel as it is the most invalidating and life-threatening complication of the disease^36^. Another interesting gene was *CREBBP*, found in family 6, which is essential for differentiation of neurons and glial cells during the embryonic cortex^37^ and tube neural development^38^. We highlight the presence of the variant in this gene together with the variant in *TSC2* gene, which has been related with the disruption of differentiation and maturation of neural precursors because its protein product regulates both cellular proliferation^39^ and migration^40^, crucial processes in HSCR. Thus, *CREBBP* and *TSC2* genes seem to have a relevant role in family 6 and also they could play an interesting role in the disorder. Therefore, the contribution of different combinations of variants in known HSCR-associated genes and other ENS candidate genes, together with variants in *FAT3* may lead to the expression of HSCR phenotype in each patient of these families. Nevertheless, as none of these gene combinations were recurrently found, nor they were tested *in vivo*, future large-scale studies or functional experiments are needed to verify their causality.

Regarding the families that did not carry rare damaging variants in *FAT3*, we highlight family 8 which presented two variants in *AHNAK* gene in heterozygosis that cosegregated with the disease. This gene was originally reported in neural crest-derived tumour cells^41^ and lately reported to be expressed in migrating neural crest cells^42^. Both *AHNAK* variants follow a recessive model of inheritance that may contribute to explain the phenotype of this family and both patients also carry a variant in *ENDRB*. We suggest that such *ENDRB* variant in this context could modulate the penetrance of variants in *AHNAK* or modify the expression of the disease in affected individuals. Finally, *DYPD* and *CNTN5* genes fitting with dominant model of inheritance in families 1 and 5, respectively, were prioritized due to its role in neurodevelopment. *DPYD* gene has been linked to neural development through a microarray analysis where it is expressed in embryonic progenitor cells^43^. *CNTN5* has been implicated in cell surface interactions during nervous system development and neurite outgrowth which could also contribute to the etiology of disease^44^.

There are some limitations in our study. False negative (FN) signal due to technology weakness is one concern for our exome sequencing. First of all, exome that includes only coding sequences is still in expansion; hence the concentration on earlier Consensus CDS is prone to FN for new functional sequences. Secondly, a small fraction of the exome (~5–10%) is poorly covered or altogether missed, largely owing to factors that are not specific to exome capture^45^. Thirdly, non-coding regions are only partially covered and completely ignored in our downstream analysis. By the reduction of sequencing cost and development of advanced analytical tool, whole genome sequencing provides a better strategy to avoid above FN issues. The results provided in our study support a notable degree of inter- and intrafamilial genetic heterogeneity. Specifically, variant sharing within a family was a necessary criterion for gene prioritization; however, this may not always be the underlying model given possible heterogeneity within each family. Different genes need to be found out to explain the differences among the patients in the same family. Incomplete penetrance and interfamilial variation are usually detected for mutations in HSCR genes.

In summary, our results have led to the identification of several new genes that likely play a role in HSCR or ENS development, although the complexity of familial HSCR cases revealed by this study highlights the current difficulties in genetic counselling.

## Methods

### HSCR families and controls

HSCR families were selected from a larger clinical database consisting of 26 families, all derived from our Department of Genetics, Reproduction and Fetal Medicine in which their probands were previously screened by Sanger sequencing for 19 HSCR candidate genes (Supplementary S3 Table). Rare coding variants found in any of these genes were checked in all family members for cosegregation pattern. A total of 8 Spanish HSCR families (Table 1) comprising 16 affected (n = 16; 12 males and 4 females) and 32 unaffected individuals could not be fully explained by previously gene screening, and they were included in this exome sequencing project. The study was carried out in accordance with the tenets of the Declaration of Helsinki and the Institutional Review Board of our institution. All experimental protocols were approved by Hospital Universitario Virgen del Rocío. Prior to their participation, written informed consent was obtained from all subjects.

In addition, data from 252 Spanish phenotyped healthy individuals that had been whole exome sequenced in the context of the Medical Genome Project, were included for comparisons^46^.

We have also compared the difference of *FAT3* burden between such public controls and 527 Spanish controls, using the same software under the same parameters (unpublished data).

### DNA library preparation and sequencing

Peripheral blood was collected and genomic DNA was isolated from current available cases and family healthy individuals, using MagNA Pure LC system (Roche, Indianapolis, IN) according to the manufacturer’s instructions. DNA samples were stored at −80 °C until used. Exome sequencing by ABI Solid 5500xl (single-end, 75 bp length, Nimblegen 2.0 capture array) was performed on the 48 individual exomes, as well as on the control group. Briefly, library preparation and exome capture were performed according to a protocol based on the Baylor College of Medicine protocol version 2.1 with several modifications. Briefly, 5 μg of input genomic DNA was sheared, end repaired, and ligated with specific adaptors. A fragment size distribution ranging from 160 bp to 180 bp after shearing and 200–250 bp after adaptor ligation was verified by Bioanalyzer (Agilent Technologies, Santa Clara, CA). The library was amplified by precapture linker-mediated polymerase chain reaction (LM-PCR) using Fast-Start High Fidelity PCR System (Roche, Indianapolis, IN). After purification, 2 μg of LM-PCR product was hybridized to V3 NimbleGen SeqCap EZ Exome libraries. After washing, amplification was performed by postcapture LM-PCR using FastStart High Fidelity PCR System (Roche). Capture enrichment was measured by qPCR according to NimbleGen protocol. The successfully captured DNA was measured by Quant-iT^TM^ PicoGreen_dsDNA reagent (Invitrogen, Carlsbad, CA) and subjected to standard sample preparation procedures for sequencing with SOLiD 5500xl platform as recommended by the manufacturer. Shortly, emulsion PCR was performed on E80 scale (about 1 billion template beads) using a concentration of 0.616 PM of enriched captured DNA. After breaking and enrichment, around 150 to 300 million templated beads were sequenced per lane on six-lane SOLiD 5500xl slides.

### Variant calling and evaluation

Raw read sequences were aligned by Bfast^47^. The aligned reads in BAM format were then pre-processed for calling with local Indel realignment, PCR duplicates removal and base quality recalibration^48^. Single nucleotide variants (SNVs) and short insertions or deletions (Indels) were called by GATK2.0^48^. Variant quality score recalibration (VQSR) and hard filtration were adopted on raw SNVs and Indels respectively. In detail, variants with low VQSR lod score (below zero), SNVs labelled as “QD < 2.0” or “MQ < 40.0” or “FS > 60.0” or “HaplotypeScore > 13.0” or “MQRankSum < −12.5” or “ReadPosRankSum < −8.0” and Indels labelled as “QD < 2.0” or “ReadPosRankSum < −20.0” or “InbreedingCoeff < −0.8” or “FS > 200.0” were excluded as low-quality variants. Individual genotypes were evaluated by genotyping quality; heterozygotes were kept only if they were supported by > 4 reads, and the ratio for alternative allele was above 0.25. Comparatively, reference or alternative homozygote was accepted if it was supported by >4 reads, and ratio for reference or alternative allele was above 0.95. After above quality control, clean variant sets from each individual were evaluated by GATK VarEval module for total number of variants, dbSNP137 coverage and Transition/Transversion (Ti/Tv) ratio. Pairwise kinship estimation was used to evaluate cross-individual relationship; this was done by PLINK using high-quality common (minor allele frequency (MAF) > 1%) and independent (linkage disequilibrium R-square < 0.2) SNPs^49^.

### Rare damaging variant filtering

All variants falling into exonic regions (hg19 Refgene) were included, and were then filtered against public databases (dbSNP137, 1000 Human Genome Project, NHLBI Exome Sequencing Project) and 252 healthy Spanish individuals using Annovar and Galaxy^50^. Only those variants with (MAF) <0.01 in each database were retained and treated as rare variants. KGGSeq was used to further exclude synonymous or non-deleterious missense variants to keep only rare damaging variants^51^. In detail, a logic model that integrates prediction scores of different programs (Polyphen2, Sift, MutationTaster, PhyloP and Likelihood ratio) was used to differentiate damaging and non-damaging variants^52^. Finally, the same variants belonging to >=2 families (out of 8 in total) were excluded as they are more likely technical artefacts given the attribute of rare damaging variants we defined.

### Analysis of rare damaging variants

Figure 2 gave the overall flowchart for pinpointing candidate genes that could be related to HSCR phenotype. Assuming that a major susceptibility gene is underlying multiple families, we searched for gene recurrence with different rare damaging variants, each of which was shared by two affected relatives in the same family. To provide statistical implication, the distribution of rare damaging variants in those recurrent genes were compared between HSCR families and 1304 background genomes (1057 1000 genome project genomes, 54 Complete Genomics genomes, 184 genomes from Danish exomes and 9 genomes from 10 Gen data) using pVAAST, a disease-gene identification tool incorporating both genetic linkage and rare variant association information^53^; in addition, gene-level burden were also compared between HSCR families and 527 MGP Spanish healthy controls using raremetal^54^,^55^. In parallel, variants shared within each family were prioritized by pedigree cosegregation. Heterozygotes with full penetrance, de novo mutations, homozygous or compound heterozygotes were considered based on a possible mode of inheritance (autosomal dominant, autosomal recessive and X-linked) for each pedigree. Biological knowledge (canonical pathways, coexpression networks and protein-protein interactions) were used to fish out remaining variants located in genes linking up with recurrent genes and genes previously implicated HSCR candidate genes; these were done by KGGSeq^51^, DAPPLE^56^, GeneMania^57^ and NetGestalt^58^. Joint effects for the genes from the same pathway/network were examined by tracing their combined cosegregation in those families not explainable by recurrent genes.

Finally, we checked the expression in ENS of our new candidate genes using Expression Atlas^59^ and The Human Protein Atlas^60^ databases.

### Validation by Sanger sequencing

Two batches of rare damaging variants were sent for wet-lab Sanger validation: the first batch covering most variants shared within each family, the second batch for interesting genes/variants linking to recurrent genes or ENS candidate genes. Each potential disease-causing variant was confirmed by Sanger sequencing, and cosegregation analyses were performed in the rest of the family members with available DNA samples. Primers for validation were designed using Primer3 (http://bioinfo.ut.ee/primer3 0.4.0/). PCR products were purified with Illustra^TM^ ExoProStar^TM^ 1-Step (GE Healthcare Life Science). Sequences were prepared with BigDye Terminator v3.1 Cycle Sequencing (Life Technologies) and finally sequenced in an automated ABI 3730 (Life Technologies).

### Gene accession numbers

*RET* (NM_020975), *PIGV* (NM_001202554), *DPYD* (NM_000110), *QTRTD1* (NM_024638), *FAT3* (NM_001008781), *TSC2* (NM_000548), *THBS4* (NM_003248), *PLAU* (NM_001145031), *FBN1* (NM_000138), *SEMA3D* (NM_152754), *NTF3* (NM_001102654), *IRAK3* (NM_007199), *KDR* (NM_002253), *GFRA1* (NM_001145453), *ZHX2* (NM_014943), *TPCN1* (NM_001143819), *AADACL4* (NM_001013630), *RPE65* (NM_000329), *HRNR* (NM_001009931), *DISP1* (NM_032890), *LYPD6* (NM_194317), *TTN* (NM_00126755), *POLQ* (NM_199420), *RHOBTB3* (NM_014899), *COL10A1* (NM_000493), *RNF148* (NM_198085), *SHOC2* (NM_007373), *AALAD2* (NM_005467), *CNTN5* (NM_014361), *B4GALNT3* (NM_173593), *PLEKHO2* (NM_025201), *PKD1L2* (NM_052892), *FKBP10* (NM_021939), *MAN2B1* (NM_000528), *PLVAP* (NM_031310), *TMPRSS15* (NM_002772), *COL6A6* (NM_001102608), *TNXB* (NM_019105), *CREBBP* (NM_004380), *TSC2* (NM_000548), *PTCH1* (NM_000264), *EDNRB* (NM_001201397), *AHNAK* (NM_001620).

### Data submission

Data are submitted to ClinVar database. Available at URL: http://www.ncbi.nlm.nih.gov/clinvar/?LinkName=orgtrack_clinvar&from_uid=505435.

## Additional Information

**How to cite this article**: Luzón-Toro, B. *et al.* Exome sequencing reveals a high genetic heterogeneity on familial Hirschsprung disease. *Sci. Rep.* **5**, 16473; doi: 10.1038/srep16473 (2015).

## Supplementary Material

Supplementary Tables

## Figures and Tables

**Table 1:** Characteristics of the patients and pre-screening results.

Family ID	Patient ID	Gender	Phenotype	Pre-screening^e^	Inheritance	RET enhancer SNP (rs2435357)
1	III.3	Male	TCA^a^	Negative	–	T/T
1	II.2	Male	NA^b^	***RET*****: c.2371T > A:p.Tyr791Asn**	Maternal	T/C
2	II.4	Female	S-HSCR^c^	Negative	–	T/C
2	II.5	Male	S-HSCR	Negative	–	T/C
3	III.1	Male	S-HSCR	***NTF3*****: c.226G > A:p.Gly76Arg**	Paternal	T/T
3	III.2	Male	L-HSCR^d^	*PHOX2B*: c.741_761del ***SEMA3D*****:c.185_186insT:p.Leu62fs**	Maternal	C/C
4	II.2	Male	NA	*PHOX2B*: c.745_765del	Maternal	T/T
4	II.8	Female	S-HSCR	Negative	–	T/C
5	I.2	Female	NA	Negative	–	C/C
5	II.1	Female	L-HSCR	*RET*: c.844G > T:p.Val282Leu	Paternal	C/C
6	II.2	Male	NA	Negative	–	T/C
6	II.1	Male	NA	Negative	–	T/C
7	III.1	Male	S-HSCR	Negative	–	T/T
7	IV.1	Male	S-HSCR	***SEMA3D*****: c.592G > A:p.Glu198Lys**	Maternal	T/C
8	II.1	Male	NA	***EDNRB*****: c.466C > T:p.Pro156Ser**	Paternal	T/T
8	II.2	Male	NA	***EDNRB*****: c.466C > T:p.Pro156Ser**	Paternal	T/T

^a^TCA = total colonic aganglionosis.

^b^NA = Not available data.

^c^S-HSCR = short segment HSCR.

^d^L-HSCR = long-segment HSCR.

^e^Damaging variants were in bold.

**Table 2:** Summary of rare damaging variants in each family.

Family ID	Patient ID	#Variants	#Variants shared (LOF)^a^	#2-Hits^b^	#X-linked	#Heterozygotes
1	II.2	154	57 (8)	0	NA^c^	3
	III.3	153				
2	II.5	130	68 (6)	0	NA	0
	II.4	155				
3	III.1	198	15 (3)	0	0	NA
	III.2	209				
4	II.2	174	10 (1)	0	NA	NA
	II.8	139				
5	II.1	135	53 (1)	0	NA	20
	I.2	149				
6	II.1	136	48 (2)	0	0	Paternal DNA not available
	II.2	134				
7	III.1	208	2 (0)	0	NA	NA
	IV.1	150				
8	II.1	179	90 (11)	1	0	0
	II.2	150				

^a^Variants present in both patients.

^b^Homozygous variants or variants forming compound heterozygotes.

^c^Not applicable.

**Table 3:** Genes recurrently mutated.

Genes	Family ID^a^	Patient and Family ID^b^	#Variants	*P*-value	LOD
*FAT3*	2, 3, 6	4: II.8, 7: III.1, 7: IV.1	6	**0,0040**	1,25
*RHOBTB3*	1, 5	0	2	0,0193	0,84
*CNTN5*	5, 8	0	2	0,1300	0,09
*TSC2*	2, 6	0	2	0,1360	0,30
*FAT4*	2, 5	3: III.1, 6: II.1	4	0,1660	0,75
*DNAH9*	2, 8	6: II.2	4	0,1830	0,47
*IGSF10*	1, 2	3: III.2	3	0,2670	0,00
*PLEC*	2, 8	0	2	0,4260	0,84
*TTN*	5, 6	4: II.2	3	0,9410	0,23

*P* value significant after Bonferroni correction is in bold.

^a^Families where both patients carry the same variant.

^b^Families with variants in only one patient

**Table 4:** Variants in *FAT3* gene found in the study.

Family	Variants	PublicDatabase_MAF^a^	ExAC_MAF (Non-Finnish CEU)	Medical Genome Project_MAF^b^	*In silico prediction (SIFT; Polyphen; Mutation_taster; logiscReg)*^c^
Fam2	c.8680G > T:p.Val2894Leu	0,000236742	3,00E-05	NA	T;B;D;Y
Fam3	c.2873T < C:p.Leu958Pro	0,000681	0,000765	0,0019	D;D;D;Y
Fam4	c.13193G > A:p.Gly4398Asp	0,002501191	0,001971	0,0037	D;D;D;Y
Fam6	c.3472A > G:p.Met1158Val	N	1,50E-05	NA	T;P;N;Y
Fam7	c.6916G > A:p.Val2306Ile	N	N	NA	T;D;D;Y
	c.6932C > G:p.Ser2311Cys	N	N	NA	D;P;D;Y

^a^Only maximum MAF across 1000 genome, dbSNP137 and ESP6500 was shown; N means variant not present in any public database.

^b^Include 252 Spanish healthy individuals; NA means not available.

^c^*In silico* prediction obtained from SIFT (T for tolerant, D for damaging), Polyphen-2 (B for benign, P for possibly damaging, D for probably damaging), Mutation taster (D for disease causing, N for polymorphism) and logistic regression model (Y for is deleterious) in KGGSeq.
