# Supplementary material for: Exome sequencing reveals a high genetic heterogeneity on familial Hirschsprung disease
Source: Sci Rep. 2015 Nov 12;5:16473. doi: 10.1038/srep16473 (PMC4642299; doi:10.1038/srep16473)
Supplement: Supplementary Tables [file srep16473-s1.doc]

**Exome sequencing reveals a high genetic heterogeneity on familial Hirschsprung disease.**

Berta Luzón-Toro^1,2¶^, Hongsheng Gui^3,4¶^, Macarena Ruiz-Ferrer^1,2^, Clara Sze-Man Tang^4,5^, Raquel M. Fernández^1,2^, Pak-Chung Sham^3,4,6,7^, Ana Torroglosa^1,2^, Paul Kwong-Hang Tam^5,7^, Laura Espino-Paisán^1^, Stacey S. Cherny^3,4,6^, Marta Bleda^2,8^, María del Valle Enguix-Riego^1,2^, Joaquín Dopazo^2,8,9^, Guillermo Antiñolo^1,2^, María-Mercé García-Barceló^5,7^, Salud Borrego^1,2*^

**Affiliations:**

^1^Department of Genetics, Reproduction and Fetal Medicine, Institute of Biomedicine of Seville (IBIS), University Hospital Virgen del Rocío/CSIC/University of Seville, Seville, Spain

^2^Centre for Biomedical Network Research on Rare Diseases (CIBERER), Spain

^3^Centre for Genomic Sciences, Li Ka Shing Faculty of Medicine, The University of Hong Kong, Hong Kong, China

^4^Department of Psychiatry, Li Ka Shing Faculty of Medicine, The University of Hong Kong, Hong Kong, China

^5^Department of Surgery, Li Ka Shing Faculty of Medicine, The University of Hong Kong, Hong Kong, China

^6^State Key Laboratory of Brain and Cognitive Sciences, Li Ka Shing Faculty of Medicine, The University of Hong Kong, Hong Kong, China

^7^Centre for Reproduction, Development, and Growth, Li Ka Shing Faculty of Medicine, The University of Hong Kong, Hong Kong, China

^8^Computational Genomics Department, Centro de Investigación Príncipe Felipe (CIPF), Valencia, Spain

^9^Functional Genomics Node, (INB) at CIPF, Valencia, Spain

^¶^Equal contribution authors

Current address for MB: Department of Medicine, University of Cambridge, School of Clinical Medicine, Addenbrooke's Hospital, Cambridge, United Kingdom

^*^Corresponding author:

Salud Borrego, MD, PhD

Department of Genetics, Reproduction and Fetal Medicine.

University Hospital Virgen del Rocío. Av Manuel Siurot s/n, 41013 Seville, Spain

Voice: + 34 955 012780/+ 34 955 014356/+ 34 955 012776

E-mail: [salud.borrego.sspa@juntadeandalucia.es](mailto:salud.borrego.sspa@juntadeandalucia.es) (SB)

**Supplementary Material**

| **S1 Table.Detailed sequencing metrics for each individual** | | | | | | | | | | |
| --- | --- | --- | --- | --- | --- | --- | --- | --- | --- | --- |
| **Family** | **Sample Name ^a^** | **Mean depth** | **% >10 fold** | **%>4 fold** | **nSNVs** | | **nINDELs** | **dbSNP137 coverage (%)** | **Ti/TvRatio ^b^** | **Kinship^c^** |
| **1** | **III.3** | 18,4 | 61,7 | 77,5 | 8178 | | 314 | 95,6 | 3,39 |  |
|  | II.1 | 28,2 | 73,4 | 83,4 | 10817 | | 551 | 94,96 | 3,5 | 0,52 |
|  | III.1 | 27,2 | 73,1 | 83,3 | 11343 | | 515 | 95,88 | 3,44 | 0,49 |
|  | III.2 | 29,2 | 75,4 | 84,9 | 11727 | | 549 | 95,4 | 3,46 | 0,48 |
|  | II.2 | 26,4 | 72,5 | 83,6 | 11380 | | 432 | 96,34 | 3,48 | 0,53 |
|  | I.1 | 26,6 | 72,9 | 83,5 | 11141 | | 402 | 96,68 | 3,45 | 0,22 |
|  | II.3 | 24,9 | 71,0 | 82,7 | 10413 | | 472 | 95,67 | 3,48 | 0,26 |
|  | II.4 | 27,7 | 72,1 | 82,2 | 10960 | | 486 | 95,82 | 3,53 | 0,20 |
| **2** | **II.5** | 25,7 | 73,2 | 84,4 | 10381 | | 519 | 95,29 | 3,52 |  |
|  | II.4 | 29,1 | 76,1 | 85,4 | 11909 | | 551 | 95,49 | 3,6 | 0,53 |
|  | I.2 | 30,1 | 76,2 | 85,3 | 12063 | | 516 | 95,89 | 3,52 | 0,51 |
|  | I.1 | 29,6 | 76,5 | 85,6 | 11784 | | 624 | 94,88 | 3,55 | 0,51 |
|  | II.1 | 25,5 | 74,8 | 85,8 | 11456 | | 522 | 95,6 | 3,52 | 0,47 |
|  | II.2 | 29,0 | 78,3 | 87,5 | 12371 | | 608 | 95,3 | 3,48 | 0,51 |
|  | II.3 | 28,7 | 78,3 | 87,9 | 12614 | | 570 | 95,47 | 3,56 | 0,51 |
| **3** | **III.1** | 47,3 | 84,1 | 88,6 | 15055 | | 299 | 98,35 | 3,44 |  |
|  | II.1 | 42,7 | 81,1 | 86,6 | 14082 | | 276 | 98,28 | 3,52 | 0,50 |
|  | II.2 | 42,2 | 81,3 | 86,7 | 14556 | | 277 | 98,48 | 3,47 | 0,53 |
|  | III.2 | 29,5 | 77,4 | 83,6 | 11736 | | 688 | 94,37 | 3,48 | 0,14 |
|  | II.4 | 29,3 | 75,9 | 87,4 | 12328 | | 488 | 96,14 | 3,51 | 0,00 |
|  | II.3 | 28,1 | 75,3 | 85,4 | 11311 | | 494 | 95,71 | 3,57 | 0,28 |
| **4** | **II.2** | 55,9 | 83,6 | 89,1 | 13609 | | 238 | 98,56 | 3,51 |  |
|  | I.1 | 55,8 | 83,5 | 88,7 | 13487 | | 241 | 98,66 | 3,45 | 0,47 |
|  | I.2 | 31,2 | 79,6 | 81,6 | 12956 | | 617 | 94,81 | 3,45 | 0,51 |
|  | II.3 | 58,8 | 75,4 | 85,3 | 12003 | | 210 | 98,85 | 3,52 | 0,50 |
|  | II.1 | 54,2 | 79,3 | 89,2 | 12808 | | 224 | 98,73 | 3,54 | 0,48 |
|  | I.4 | 22,1 | 68,9 | 82,9 | 8965 | | 407 | 93,8 | 3,34 | 0,25 |
|  | II.5 | 27,6 | 74,1 | 84,5 | 11021 | | 551 | 95,14 | 3,49 | 0,10 |
|  | II.7 | 28,8 | 73,6 | 83,4 | 11070 | | 472 | 95,92 | 3,51 | 0,14 |
|  | II.4 | 30,0 | 76,8 | 86,0 | | 12008 | 602 | 95,13 | 3,46 | 0,13 |
|  | II.8 | 55,4 | 75,9 | 81,7 | | 11838 | 191 | 98,63 | 3,42 | 0,10 |
|  | I.5 | 53,4 | 79,0 | 85,4 | | 12593 | 213 | 98,47 | 3,46 | 0,00 |
| **5** | **II.1** | 24,7 | 72,3 | 83,7 | | 11474 | 362 | 96,86 | 3,51 |  |
|  | I.2 | 28,9 | 73,7 | 82,0 | | 11357 | 434 | 96,16 | 3,46 | 0,50 |
|  | I.1 | 22,6 | 69,6 | 86,0 | | 10667 | 377 | 96,34 | 3,48 | 0,51 |
| **6** | **II.2** | 28,5 | 75,5 | 86,0 | | 11418 | 494 | 95,78 | 3,53 |  |
|  | II.1 | 29,1 | 76,7 | 86,5 | | 12316 | 524 | 95,83 | 3,56 | 0,55 |
|  | I.1 | 27,0 | 73,9 | 85,2 | | 11504 | 407 | 96,69 | 3,62 | 0,52 |
| **7** | **III.1** | 31,3 | 78,6 | 87,7 | | 12647 | 648 | 94,91 | 3,47 |  |
|  | II.2 | 30,6 | 78,8 | 88,1 | | 13171 | 499 | 96,26 | 3,46 | 0,51 |
|  | II.1 | 30,4 | 77,2 | 86,7 | | 12505 | 505 | 96,25 | 3,5 | 0,50 |
|  | IV.1 | 27,6 | 73,7 | 83,9 | | 11681 | 437 | 96,43 | 3,45 | 0,00 |
|  | I.1 | 31,0 | 78,7 | 87,7 | | 13013 | 406 | 96,82 | 3,47 | 0,00 |
| **8** | **II.1** | 53,7 | 87,5 | 91,0 | | 15083 | 285 | 98,75 | 3,35 |  |
|  | II.2 | 49,1 | 85,9 | 90,2 | | 14599 | 272 | 98,90 | 3,37 | 0,53 |
|  | II.3 | 55,0 | 82,9 | 88,0 | | 13993 | 276 | 98,87 | 3,40 | 0,54 |
|  | I.1 | 53,2 | 87,3 | 90,9 | | 14845 | 278 | 98,89 | 3,38 | 0,51 |
|  | I.2 | 53,0 | 87,7 | 91,2 | | 15063 | 286 | 98,73 | 3,33 | 0,51 |
|  | **Average** | 34,9 | 76,9 | 85,7 | | 12194 | 429 | 96,56 | 3,48 | 0,00 |
| ^a^ proband in each family is in bold; ^b^ Ti/Tv means transition versus transversion sites; ^c^ only kinship with proband in each family is shown; average kinship value is calculated from unrelated cases from different families. | | | | | | | | | | |

| **S2 Table. Cosegregation analysis of prioritized rare damaging mutations in each family** | | | | | | | | | | | | | | | | | | |
| --- | --- | --- | --- | --- | --- | --- | --- | --- | --- | --- | --- | --- | --- | --- | --- | --- | --- | --- |
| **Family** | **Genes^a^** | **RefSeq** | **Variants** | **PublicDatabase_MAF** | **ExAC MAF (Non-Finnish CEU)** | **Medical Genome Project_MAF** | **Genotypes^c^** | | | | | | | | | | | **Annotation^d^** |
| **Fam1** |  |  |  |  |  |  | **III.3** | II.1 | III.1 | III.2 | **II.2** | I.1 | II.3 | II.4 |  |  |  |  |
|  | *RET* | NM_020975 | c.2371T>A:p.Tyr791Asn | N ^b^ | 0,00003037 | N | 0/0 | 0/0 | 0/1 | 0/1 | 0/1 | 0/1 | 0/0 | 0/1 |  |  |  |  |
|  | ***PIGV*** | NM_001202554 | c.364C>T:p.Leu122Phe | N | N | N | 0/1 | 0/0 | 0/0 | 0/0 | 0/1 | 0/0 | 0/0 | 0/0 |  |  |  |  |
|  | ***DPYD*** | NM_000110 | c.1905+1G>A | 0,005813954 | 0,005833 | N | 0/1 | 0/0 | 0/0 | 0/0 | 0/1 | 0/0 | 0/0 | 0/0 |  |  |  |  |
|  | ***QTRTD1*** | NM_024638 | c.77_78insA:p.His26fs | N | N | N | 0/1 | 0/0 | 0/0 | 0/0 | 0/1 | 0/0 | 0/0 | 0/0 |  |  |  |  |
| **Fam2** |  |  |  |  |  |  | **II.4** | I.2 | I.1 | II.1 | II.2 | II.3 | **II.5** |  |  |  |  |  |
|  | *FAT3* | NM_001008781 | c.8680G>T:p.Val2894Leu | 0,000236742 | 0,00002999 | N | 0/1 | 0/1 | 0/0 | 0/1 | 0/0 | 0/1 | 0/1 |  |  |  |  |  |
|  | *TSC2* | NM_000548 | c.3889G>A:p.Ala1297Thr (rs45517319) | 0,004084968 | 0,009104 | N | 0/1 | 0/1 | 0/0 | 0/0 | 0/0 | 0/1 | 0/1 |  |  |  |  | REACTOME_SIGNALING_BY_PDGF |
|  | *THBS4* | NM_003248 | c.1885C>T:p.Gln629* | N | 0 | N | 0/1 | 0/0 | 0/1 | 0/0 | 0/0 | 0/0 | 0/1 |  |  |  |  | REACTOME_SIGNALING_BY_PDGF |
|  | *PLAU* | NM_001145031 | c.121G>A:p.Gly41Arg | 0,008953488 | 0,008846 | 0,0019 | 0/1 | 0/1 | 0/0 | 0/1 | 0/0 | 0/1 | 0/1 |  |  |  |  | PID_INTEGRIN5_PATHWAY |
|  | *FBN1* | NM_000138 | c.1547G>A:p.Arg516Gln | N | 0 | N | 0/1 | 0/0 | 0/1 | 0/0 | 0/0 | 0/0 | 0/1 |  |  |  |  | PID_INTEGRIN5_PATHWAY |
| **Fam3** |  |  |  |  |  |  | **III.1** | II.1 | II.2 | **III.2** | II.4 | II.3 |  |  |  |  |  |  |
|  | *FAT3* | NM_001008781 | c.2873T<C:p.Leu958Pro | 0,000681 | 0,0007647 | 0,0019 | 0/1 | 0/0 | 0/1 | 0/1 | 0/0 | 0/1 |  |  |  |  |  |  |
|  | *SEMA3D* | NM_152754 | c.185_186insT:p.Leu62fs | N | N | N | 0/0 | 0/0 | 0/0 | 0/1 | 0/0 | 0/1 |  |  |  |  |  |  |
|  | *NTF3* | NM_001102654 | c.226G>A:p.Gly76Arg | N | 0,0002424 | 0,0019 | 0/1 | 0/1 | 0/0 | 0/0 | 0/0 | 0/0 |  |  |  |  |  | PID_SHP2_PATHWAY, KEGG_NEUROTROPHIN_SIGNALING_PATHWAY |
|  | *IRAK3* | NM_007199 | c.1461delC:p.Asn487fs | 0,000848074 | 0,0008392 | N | 0/1 | 0/0 | 0/1 | 0/1 | 0/0 | 0/1 |  |  |  |  |  | KEGG_NEUROTROPHIN_SIGNALING_PATHWAY |
|  | *KDR* | NM_002253 | c.2066C>T:p.Thr689Met | 0,004069767 | 0,003043 | 0,0056 | 0/0 | 0/0 | 0/0 | 0/1 | 0/1 | 0/0 |  |  |  |  |  | PID_SHP2_PATHWAY |
| **Fam4** |  |  |  |  |  |  | **II.8** | I.5 | I.4 | II.3 | **II.2** | II.1 | I.1 | I.2 | II.5 | II.7 | II.4 |  |
|  | *FAT3* | NM_001008781 | c.13193G>A:p.Gly4398Asp | 0,002501191 | 0,001971 | 0,0037 | 0/1 | 0/1 | 0/0 | 0/0 | 0/0 | 0/0 | 0/0 | 0/0 | 0/0 | 0/0 | 0/0 |  |
|  | *GFRA1* | NM_001145453 | c.764T>C:p.Leu255Pro | N | N | N | 0/1 | 0/0 | 0/1 | 0/0 | 0/1 | 0/0 | 0/1 | 0/0 | 0/0 | 0/0 | 0/0 | GFRA1<->RET (STRING) |
|  | *ZHX2* | NM_014943 | c.1682G>A:p.Arg561Gln | 0,001074 | 0,0006804 | N | 0/1 | 0/1 | 0/0 | 0/0 | 0/0 | 0/0 | 0/0 | 0/0 | 0/0 | 0/0 | 0/1 | ZHX2<->RET (STRING) |
|  | *TPCN1* | NM_001143819 | c.1369G>T:p.Ala457Ser | 0,001162791 | 0,001743 | N | 0/0 | 0/0 | 0/0 | 0/1 | 0/1 | 0/0 | 0/0 | 0/1 | 0/0 | 0/0 | 0/0 | TPCN1<->RET (STRING) |
| **Fam5** |  |  |  |  |  |  | **I.2** | I.1 | **II.1** |  |  |  |  |  |  |  |  |  |
|  | ***AADACL4*** | NM_001013630 | c.974C>G:p.Pro325Arg | N | 0,000225 | N | 0/1 | 0/0 | 0/1 |  |  |  |  |  |  |  |  |  |
|  | ***RPE65*** | NM_000329 | c.1220T>C:p.Val407Ala (rs62636297) | N | 0 | N | 0/1 | 0/0 | 0/1 |  |  |  |  |  |  |  |  |  |
|  | ***HRNR*** | NM_001009931 | c.325C>A:p.Gln109Lys | 0,000348837 | 0,00012 | N | 0/1 | 0/0 | 0/1 |  |  |  |  |  |  |  |  |  |
|  | ***DISP1*** | NM_032890 | c.1640G>A:p.Arg547His | N | 0,00001499 | N | 0/1 | 0/0 | 0/1 |  |  |  |  |  |  |  |  |  |
|  | ***LYPD6*** | NM_194317 | c.143A>C:p.Lys48Thr (rs138915968) | 0,000697674 | 0,001215 | N | 0/1 | 0/0 | 0/1 |  |  |  |  |  |  |  |  |  |
|  | ***TTN*** | NM_001267550 | c.83516G>A:p.Arg27839Gln | 0,000366479 | 0,0004527 | N | 0/1 | 0/0 | 0/1 |  |  |  |  |  |  |  |  |  |
|  | ***POLQ*** | NM_199420 | c.7259A>G:p.Tyr2420Cys (rs150364457) | 0,000465116 | 0,0004951 | N | 0/1 | 0/0 | 0/1 |  |  |  |  |  |  |  |  |  |
|  | ***RHOBTB3*** | NM_014899 | c.356T>C:p.Ile119Thr (rs144735826) | 0,001046512 | 0,000435 | N | 0/1 | 0/0 | 0/1 |  |  |  |  |  |  |  |  |  |
|  | ***COL10A1*** | NM_000493 | c.172G>A:p.Glu58Lys | 0,000116279 | 0 | N | 0/1 | 0/0 | 0/1 |  |  |  |  |  |  |  |  |  |
|  | ***RNF148*** | NM_198085 | c.128A>C:p.Asn43Thr | N | N | N | 0/1 | 0/0 | 0/1 |  |  |  |  |  |  |  |  |  |
|  | ***SHOC2*** | NM_007373 | c.770T>C:p.Ile257Thr | N | N | N | 0/1 | 0/0 | 0/1 |  |  |  |  |  |  |  |  |  |
|  | ***NAALAD2*** | NM_005467 | c.1499C>T:p.Pro500Leu | N | N | N | 0/1 | 0/0 | 0/1 |  |  |  |  |  |  |  |  |  |
|  | ***CNTN5*** | NM_014361 | c.3016G>A:p.Val1006Ile | N | 0 | N | 0/1 | 0/0 | 0/1 |  |  |  |  |  |  |  |  |  |
|  | ***B4GALNT3*** | NM_173593 | c.2597A>G:p.Asp866Gly | N | 0 | N | 0/1 | 0/0 | 0/1 |  |  |  |  |  |  |  |  |  |
|  | ***PLEKHO2*** | NM_025201 | c.152A>G:p.Tyr51Cys | N | 0,00007494 | N | 0/1 | 0/0 | 0/1 |  |  |  |  |  |  |  |  |  |
|  | ***PKD1L2*** | NM_052892 | c.6189C>G:p.Asp2063Glu | 0,000595948 | 0,0002552 | N | 0/1 | 0/0 | 0/1 |  |  |  |  |  |  |  |  |  |
|  | ***FKBP10*** | NM_021939 | c.923C>T:p.Ser308Phe | 0,000232558 | 0,0001663 | N | 0/1 | 0/0 | 0/1 |  |  |  |  |  |  |  |  |  |
|  | ***MAN2B1*** | NM_000528 | c.2992C>T:p.Arg998Cys | N | 0,00004496 | N | 0/1 | 0/0 | 0/1 |  |  |  |  |  |  |  |  |  |
|  | ***PLVAP*** | NM_031310 | c.280G>A:p.Ala94Thr | N | 0,00003006 | N | 0/1 | 0/0 | 0/1 |  |  |  |  |  |  |  |  |  |
|  | ***TMPRSS15*** | NM_002772 | c.2875T>A:p.Tyr959Asn | N | N | N | 0/1 | 0/0 | 0/1 |  |  |  |  |  |  |  |  |  |
| **Fam6** |  |  |  |  |  |  | **II.2** | **II.1** | I.1 |  |  |  |  |  |  |  |  |  |
|  | *FAT3* | NM_001008781 | c.3472A>G:p.Met1158Val | N | 0,00001502 | N | 0/1 | 0/1 | 0/0 |  |  |  |  |  |  |  |  |  |
|  | *COL6A6* | NM_001102608 | c.6497T>C:p.Ile2166Thr | N | 0,00003035 | N | 0/1 | 0/1 | 0/0 |  |  |  |  |  |  |  |  | KEGG_ECM_RECEPTOR_INTERACTION |
|  | *TNXB* | NM_019105 | c.2170C>T:p.Arg724Cys | 0,002001413 | 0,001927 | 0,0019 | 0/1 | 0/1 | 0/1 |  |  |  |  |  |  |  |  | KEGG_ECM_RECEPTOR_INTERACTION |
|  | *CREBBP* | NM_004380 | c.7210G>A:p.Glu2404Lys | N | N | N | 0/1 | 0/1 | 0/0 |  |  |  |  |  |  |  |  | PID_P53DOWNSTREAMPATHWAY |
|  | *TSC2* | NM_000548 | c.1318G>A:p.Gly440Ser (rs45484298) | 0,001627907 | 0,00118 | 0,0019 | 0/1 | 0/1 | 0/1 |  |  |  |  |  |  |  |  | PID_P53DOWNSTREAMPATHWAY |
| **Fam7** |  |  |  |  |  |  | **III.1** | II.2 | II.1 | **IV.1** | I.1 |  |  |  |  |  |  |  |
|  | *FAT3* | NM_001008781 | c.6916G>A:p.Val2306Ile | N | N | N | 0/1 | 0/0 | 0/1 | 0/0 | 0/0 |  |  |  |  |  |  |  |
|  | *FAT3* | NM_001008781 | c.6932C>G:p.Ser2311Cys | N | N | N | 0/0 | 0/0 | 0/0 | 0/1 | 0/0 |  |  |  |  |  |  |  |
|  | *SEMA3D* | NM_152754 | c.592G>A:p.Glu198Lys | N | 0,00001512 | N | 0/0 | 0/0 | 0/0 | 0/1 | 0/1 |  |  |  |  |  |  | KEGG_AXON_GUIDANCE |
|  | *PTCH1* | NM_000264 | c.1306G>A:p.Asp436Asn | 0,0005 | 0,001064 | 0,0056 | 0/1 | 0/1 | 0/0 | 0/0 | 0/0 |  |  |  |  |  |  | PTCH1<->RET |
| **Fam8** |  |  |  |  |  |  | **II.1** | **II.2** | II.3 | I.1 | I.2 |  |  |  |  |  |  |  |
|  | *EDNRB* | NM_001201397 | c.736C>T:p.Pro246Ser | N | N | NA | 0/1 | 0/1 | 0/1 | 0/1 | 0/0 |  |  |  |  |  |  |  |
|  | ***AHNAK*** | NM_001620 | c.7562C>G:p.Pro2521Arg | 0,001163061 | 0,000944 | NA | 0/1 | 0/1 | 0/1 | 0/1 | 0/0 |  |  |  |  |  |  |  |
|  | ***AHNAK*** | NM_001620 | c.6539A>G:p.Lys2180Arg | N | 0,00001498 | NA | 0/1 | 0/1 | 0/0 | 0/0 | 0/1 |  |  |  |  |  |  |  |
| a genes fully following pedigree cosegregation are in bold; | | | | | | | | | | | | | | | | | | |
| b Only maxium maf in 1000Genome, dbSNP or ESP was given; N means variant not present in any public database. | | | | | | | | | | | | | | | | | | |
| c affected patients are marked as red and in bold; | | | | | | | | | | | | | | | | | | |
| d bioinformatics annotation is from protein-protein interaction database (STRING) and canonical pathway databases (Reactome, KEGG, PID). | | | | | | | | | | | | | | | | | | |

| **S3 Table: Characteristics of 19 ENS-related HSCR candidate genes previously screened in Spanish families** | | | |  |  |
| --- | --- | --- | --- | --- | --- |
| **Pathways** | **Gene** | **Gene name** | **Type of gene product** | **Chromosome** | **Gene length (kb)** |
| Ret signaling | *RET* | Ret proto-oncogene | Transmembrane receptor | 10q11 | 53,2 |
| Ret signaling | *GDNF* | Glial cell derived neurotrophic factor | Neurotrophic factor | 5p13 | 27,0 |
| Ret signaling | *NRTN* | Neurturin | Neurotrophic factor | 19p13 | 4,5 |
| Ret signaling | *ARTN* | Artemin | Neurotrophic factor | 1p34.1 | 3,9 |
| Ret signaling | *PSPN* | Persephin | Neurotrophic factor | 19p13.3 | 0,6 |
| Ret signaling | *GFRA4* | GDNF family receptor alpha 4 | Cell surface co-receptor | 20p13 | 4,1 |
| Ednrb signaling | *EDNRB* | Endothelin receptor type B | Transmembrane receptor | 13q22 | 80,0 |
| Ednrb signaling | *EDN3* | Endothelin 3 | Vasoactive peptide | 20q13 | 25,6 |
| Semophorin signaling | *SEMA3A* | Sema domain, immunoglobulin domain (Ig), short basic domain, secreted, (semaphorin) 3A | Secreted protein | 7p12 | 236,6 |
| Semophorin signaling | *SEMA3D* | Sema domain, immunoglobulin domain (Ig), short basic domain, secreted, (semaphorin) 3D | Secreted protein | 7q21.11 | 191,6 |
| Neuregulin signaling | *NRG1* | Neuregulin 1 | Signaling protein | 8p12 | 1125,0 |
| Prokineticin signaling | *PROK1* | Prokineticin 1 | Growth factor | 1p13 | 6,2 |
| Prokineticin signaling | *PROK2* | Prokineticin 2 | Growth factor | 3p13 | 13,6 |
| Prokineticin signaling | *PROKR1* | Prokineticin receptor 1 | Transmembrane receptor | 2p14 | 122,0 |
| Prokineticin signaling | *PROKR2* | Prokineticin receptor 2 | Transmembrane receptor | 20p12 | 12,4 |
| Ntrk signaling | *NTRK3* | Neurotrophic tyrosine kinase, receptor, type 3 | Transmembrane receptor | 15q25 | 379,4 |
| Ntrk signaling | *NTF3* | Neurotrophin 3 | Neurotrophic factor | 12p13 | 91,6 |
| Others | *PHOX2B* | Paired-like homeobox 2b | Transcription factor | 4p13 | 4,9 |
| Others | *SOX10* | SRY (sex determining region Y)-box 10 | Transcription factor | 22q13 | 15,0 |
